# What are barriers and facilitators for implementation of music interventions for minor surgical procedures in general practice in the Netherlands? A qualitative study

**DOI:** 10.1136/bmjopen-2025-114312

**Published:** 2026-07-15

**Authors:** Milan van Engelen, Jorrit G Verhoeven, Erwin Ista, Dieuwke Schiphof, Johannes Jeekel, Markus Klimek

**Affiliations:** 1 Department of Anaesthesiology, Erasmus MC University Medical Center Rotterdam, Rotterdam, The Netherlands; 2 Department of Neuroscience, Erasmus MC University Medical Center Rotterdam, Rotterdam, The Netherlands; 3 Department of Internal Medicine, Division of Nursing Science, Erasmus MC University Medical Center Rotterdam, Rotterdam, The Netherlands; 4 Sophia Children’s Hospital, Department of Neonatal and Pediatric Intensive Care, Division of Pediatric Intensive Care, Erasmus MC University Medical Center Rotterdam, Rotterdam, The Netherlands; 5 Department of General Practice, Erasmus MC University Medical Center Rotterdam, Rotterdam, The Netherlands

**Keywords:** General Practice, Implementation Science, COMPLEMENTARY MEDICINE

## Abstract

**Abstract:**

**Objective:**

This study aimed to identify barriers and facilitators affecting implementation of perioperative music interventions in general practice.

**Design:**

A qualitative implementation study using semistructured interviews. The updated Consolidated Framework for Implementation Research (CFIR) was used to guide data collection and analysis. The domains used were: innovation, outer setting, inner setting and individuals. Primary outcomes were barriers and facilitators for the implementation of perioperative music interventions in general practice.

**Setting:**

General practices in the Netherlands.

**Participants:**

Dutch general practitioners.

**Results:**

15 participants were included, among which 11 general practice owners, one salaried general practitioner, two locum general practitioners and one general practitioner in training. In total, 19 key barriers and 34 key facilitators were identified. For the innovation domain, a lack of research conducted in primary care and low awareness of the intervention were considered barriers whereas user-friendliness and low costs were seen as facilitators. For the outer setting domain, no barriers were identified and possibilities for external financing, inclusion in clinical guidelines and patient and media pressure were considered facilitators. For the inner setting, the lack of readily accessible information aimed at primary care was seen as a barrier, whereas a shared belief in minimising perioperative pain and anxiety was seen as a facilitator. For the individuals domain, a pre-existing heavy workload and understaffing were seen as barriers, whereas the autonomous role of general practitioners was seen as a facilitator.

**Conclusions:**

Several key barriers and facilitators for implementation of perioperative music interventions in general practice were identified. A lack of research performed in the setting of primary care of the efficacy of the music intervention and low awareness among general practitioners were examples of barriers. Notable facilitators were user-friendliness, low-costs and the autonomous position of the general practitioner. Implementation of music intervention by general practitioners seems feasible, yet there is still room for more research to be performed in the specific context of primary care.

**Strengths and limitations of this study**

- 15 general practitioners (in training) from different practices were interviewed regarding their views on implementation of music interventions.
- This study uses a validated framework to comprehensively categorise facilitators and barriers of implementation, the Consolidated Framework for Implementation Research-framework.
- Volunteer bias may have influenced the outcome of the results and limit generalisability.

## Introduction

 Music has been used for millennia to achieve certain clinical benefits. Plato believed music could heal the soul, and Bach composed the Goldberg Variations to alleviate a Russian ambassador’s insomnia. Evan O’Neill Kane was the first physician to use recorded music played through a phonograph in the operation theatre to ameliorate patients’ anxiety in 1907.^1^ Modern science has found empirical evidence for these ‘music interventions’. The analgesic effect of music interventions has been studied very well and a number of possible causal explanations have been put forward.^2^ Music has been suggested to function as a distractor from the pain experience. Another explanation is that music can elicit emotions that alleviate the pain experience.^3^ Functional MRI scans have shown that listening to favourite music while undergoing a painful stimulus activates pain modulating areas of the brain.^4^

In clinical research, a music intervention is often a defined ‘medicine’ with features set by the researchers. For example, in a study to investigate whether music interventions could reduce the incidence of delirium in patients undergoing brain surgery they were instructed to listen to their preferred music using headphones for 30 min periods, before, and after surgery, and during the procedure itself.^5^ Clinical studies have shown that music interventions are effective in reducing anxiety, stress and pain associated with surgical procedures in both adults and children and for a variety of procedures.^6^,^11^ Furthermore, perioperative music interventions may reduce the need for postoperative analgesia and increase patient satisfaction.^8 10 12^

Despite these results, implementation efforts in hospital care settings have resulted in suboptimal adherence rates, with only 29%–53% of all eligible surgical patients actually receiving music interventions as part of their perioperative care despite national guidelines promoting it.^913^,^15^ Patient motivation is not thought to be the main issue as a large survey involving 33 629 participants across 20 countries found that 86.5% would like to listen to music when experiencing pain in the healthcare setting.^16^ This has prompted an investigation into potential strategies for improvement of implementation of music interventions.

In the Netherlands, minor surgical procedures are also performed by primary care physicians (ie, general practitioners (GPs)) in 32.7 per 1000 registered patients.^17^ Surgical procedures performed by GPs include (intra-articular) injections, insertion of intrauterine devices, excision of skin lesions and partial nail extractions. Depending on the nature of the procedure, these are performed with local anaesthetic techniques and thus on fully conscious patients. Consequently, while these minor surgical procedures are less complex than major procedures involving general anaesthesia, patients may experience anxiety, stress and/or pain not only preoperatively or postoperatively, but also intraoperatively.

This opens the field for implementation of perioperative music interventions in general practice. However, a significant research gap exists pertaining to the identification of barriers and facilitators influencing the implementation process in general practice situations. In the Dutch healthcare system, GPs function as gatekeepers to other types of healthcare such as hospital care medical specialists.^17^ General practice therefore provides an accessible, first encounter with a physician for most health problems.^17^ Primary healthcare is provided by a small team of physicians and supporting staff to a regular patient population, with up to 78% of patients consulting their general practice at least once per year and an average of five consultations per patient per year.^17^ This distinction between general practice and hospital care, combined with the different nature of the surgical procedures performed, warrants a targeted assessment of contextual factors in general practice situations to successfully implement perioperative music interventions in primary care. The study of Kakar *et al*^18^ identified barriers and facilitators for implementation of perioperative music interventions in the hospital setting using the Consolidated Framework for Implementation Research (CFIR), a framework designed for systematic classification of contextual factors in implementation research.^19^ They found that lack of knowledge regarding the intervention and an existing work culture that hindered decision-making were seen as barriers for implementation. Given the fact of difference in setting and type of surgical interventions, we hypothesised that the barriers and facilitators identified in general practice situations would differ from those identified in a hospital setting.

## Methods

### Study design

The research question for this implementation study is: what are barriers and facilitators for implementation of music interventions for minor surgical procedures in primary care as perceived by GPs, using the Updated CFIR?^20^ We sought to answer that question through an explorative, qualitative study carried out in the Netherlands among GPs and GPs in training using individual semistructured interviews. The interviews were carried out from August 2024 to October 2024. The Consolidated criteria for reporting qualitative research (COREQ) checklist was used to comprehensively report the study’s methodology and findings (see online supplemental file 3).^21^

### Study population and recruitment

Participants were recruited via a composite approach. Initially, general practices in the greater Rotterdam area were approached via phone contact and email. Second, announcements were placed locally in GP emergency posts in the greater Rotterdam area, which are alternately staffed by various collaborating GPs. Subsequently, to achieve full sample size and expand the pool of potential participants, social media channels of the research group were used to extend the reach for potential participants to the Netherlands as a whole. The announcements and social media posts included a web link to a contact form. Following the voluntary registration of contact details, potential participants were approached via phone and/or email. Comprehensive written study details were provided to all participants via email.

Inclusion criteria were: (1) actively employed GPs or GPs in training and (2) performing minor surgical procedures, for instance, the excision of skin lesions or partial nail extractions. No exclusion criteria were established. The study wished to include participants from separate, variously sized general practices to ensure extensive coverage of the contextual factors and to increase independency of the findings. However, no further selection was made to accomplish this aim. Based on literature and consensus a sample size of 15 participants was expected to have sufficient information power and achieve data saturation for this study.^22 23^

### Consolidated Framework for Implementation Research

In this study, the updated CFIR was used to guide both data collection and data analysis, by providing a framework to systematically assess and classify barriers and facilitators perceived by the participants.^20^ CFIR comprised of five domains: (1) the innovation domain; (2) the outer setting domain; (3) the inner setting domain; (4) the individuals domain and (5) the implementation process domain.^20^ The fifth domain, regarding the barriers and facilitators within the implementation process itself, was not studied in the current explorative design and remains reserved for future evaluative research. The four CFIR domains examined in this study comprise a total of 39 constructs and 13 additional subconstructs.^20^ See table 1 for a brief explanation of the different domains.

**Table 1:** The CFIR framework^20^

CFIR framework*	Definition	In this study
1: Innovation domain	The ‘thing’ being implemented	Periprocedural music intervention
2: Outer setting domain	The setting in which the Inner setting exists	Primary healthcare in the Netherlands, national and regional collaborations
3: Inner setting domain	The setting in which the innovation is implemented	General practices
4: Individuals domain	The roles and characteristics of individuals	General practitioners (in training), supporting staff

* The fifth domain, implementation process domain, was not under investigation in this study.

CFIR, Consolidated framework for implementation research.

### Data collection: interview guide development

Participants were interviewed using a semistructured interview guide. Development of the interview guide consisted of four iterations of drafts, which were revised by the investigators to ensure content integrity and validity. The guide comprised three parts, totalling 39 questions. The interview guide is available as online supplemental file 1.

The first part of the interview guide examined demographic data of the participants (age, sex, work experience, employment type (practice owner, salaried GP, locum GP, GP in training), general practice size (ie, the number of physicians employed at the participant’s place of work, including GPs in training) and prior utilisation of perioperative music interventions).

The second part of the interview guide investigated the barriers and facilitators for implementation of perioperative music interventions in general practice as perceived by the participants. Questions were based on a selection of the constructs provided in the CFIR framework.^20^ Constructs of interest were selected from the four examined CFIR domains by investigator consensus, as not all constructs were considered equally informative in the context of implementation of perioperative music interventions. A question was added to each domain within the interview guide to allow for unexpected findings.

The third part of the interview guide assessed the willingness among participants to implement perioperative music interventions in general practice and whether participants believed that music interventions should be part of standard perioperative care in general practice. Reasons for or against implementation were also discussed.

### Data collection: interview assessment

Data were collected through interviews with individual participants using the aforementioned guide. The interviews were conducted in person, via phone call or via an online video call by a single investigator (MvE) to ensure consistency of the collected data. The investigator was a medical master’s student without prior involvement in music intervention research, limiting a priori assumptions or strongly held beliefs. Interviews lasted for 45–75 min, providing time for discussion, reflection and input from the perspective of the participant. The interviews were audiotaped for subsequent transcription.

### Data-analysis

The audiotaped interviews were transcribed verbatim into full-text qualitative data. Personal names and locations were anonymised. The full-text data were analysed using thematic content analysis in a deductive approach according to Braun and Clarke’s six-phase guide for thematic analysis in qualitative research.^24^ Initially, the barriers and facilitators as expressed by the participants were identified into codes. Subsequently, all established codes were classified into previously established themes using the CFIR (ie, the constructs and domains). Descriptive statistics were used to present demographic data. Numerical data (participant age, work experience and general practice size) were presented as medians and IQRs. Categorical data (participant employment type, sex and prior utilisation of music interventions) were presented as percentages. Qualitative data were analysed using NVivo, V.15,^25^ and quantitative data were analysed using Microsoft Excel, V.2402.^26^

### Outcomes

The primary outcomes of this study were the barriers and facilitators for implementation of perioperative music interventions in general practice, and the corresponding constructs according to the Updated CFIR. Secondary outcomes were willingness to implement perioperative music interventions in general practice and belief that music interventions should be part of standard perioperative care.

### Patient and public involvement

None.

## Results

### Participant baseline characteristics

15 participants from separate general practices were included in the study, of which 11 (73.3%) were general practice owners, one (6.7%) was a salaried GP, two (13.3%) were employed as locum GP and one (6.7%) was a GP in training. Median age was 55 years (IQR 15). 33.3% of participants identified as male, and 66.7% identified as female. Nine participants (60.0%) previously used music in their general practice, of which six (40.0%) previously used music perioperatively (eg, on patient request). After 13 interviews, no new themes were identified and data saturation was achieved after 15 interviews. There was no observable difference in attitude towards barriers and facilitators based on region. The baseline characteristics of the participants are further presented in table 2.

**Table 2:** Baseline characteristics of the participants

Characteristics	n=15
Age, years, median (IQR)	55 (15)
Work experience, years, median (IQR)	21 (17.5)
Number of physicians in general practice, n, median (IQR)	5 (4)
Sex, n (%)	
Male	5 (33.3)
Female	10 (66.7)
Employment type, n (%)	
General practice owner	11 (73.3)
Salaried general practitioner	1 (6.7)
Locum general practitioner	2 (13.3)
General practitioner in training	1 (6.7)
Prior utilisation of music in general practice, n (%)	
No	6 (40.0)
Yes	9 (60.0)

n, number.

### Barriers and facilitators

Across the 39 constructs and 13 subconstructs within the four examined domains of CFIR, a total of 39 barriers, 60 facilitators and two additional neutral factors were identified, which could not be clearly categorised as either barrier or facilitator. Table 3 provides an overview of the key identified barriers, facilitators and neutral factors.

**Table 3:** Key barriers and facilitators as perceived by the participants and categorised according to the Updated Consolidated Framework for Implementation Research (CFIR)

CFIR domain	Construct	Codes	Determinant
1. Innovation domain	Evidence base	Insufficient prior knowledge	Barrier
		Lack of research conducted in primary healthcare	Barrier
	Relative advantage	Preference for non-pharmacological interventions	Facilitator
		Standard treatment rarely includes additional medication prescriptions, is effective and/or irreplaceable	Barrier
	Complexity	User-friendly and not difficult to install	Facilitator
	Design	Communication impaired by headphones	Barrier
		Open attitude of general practitioners towards musical content of the patient’s preference, except extreme genres	Facilitator
		Communication impaired by music loudness	Barrier
		Lack of space and time to provide intervention prior to and after the procedure	Barrier
		Patients receptive to lifestyle advice, such as listening to music	Facilitator
	Cost	Affordable equipment	Facilitator
		License to use copyrighted music	Barrier
2. Outer setting domain	Partnerships and connections	Regional collaborations provide reach, education, funding, facilitation and contact with health insurance providers	Facilitator
		National collaborations provide reach, education, guidelines and represent interests of general practitioners	Facilitator
		Academic centres provide reach and education	Facilitator
	Policies and laws	Existing guideline aimed at secondary healthcare	Facilitator
		Development of guideline aimed at general practitioners	Facilitator
	Financing	External financing (eg, equipment, financial compensation)	Facilitator
	Societal pressure	Pressure exerted by patients and patient organisations	Facilitator
		Media pressure (eg, conventional media, social media)	Facilitator
	Market pressure	Positive peer pressure provides inspiration	Facilitator
		Indifference among general practitioners towards negative peer pressure	Barrier
3. Inner setting	Physical infrastructure	Inadequate noise-insulating properties	Barrier
domain	Communications	Possibilities for frequent peer consultation and discussion	Facilitator
		Short lines of communication due to smaller team size	Facilitator
	Recipient centredness	Shared belief on minimising patient anxiety and pain	Facilitator
	Tension for change	Reduced patient anxiety and pain due to minor, brief and less complex surgical procedures	Barrier
		Reduced patient anxiety due to communication with physician	Barrier
		Higher levels of anxiety and pain in patients undergoing insertion of intrauterine devices and partial nail extractions respectively	Facilitator
	Compatibility	Improves concentration and functioning of operating physician	Facilitator
		Limited time per consultation for minor surgical procedures	Barrier
		Diagnosing consult and separate scheduled surgical consult	Facilitator
	Relative priority	Involvement in multitude of implementation projects	Barrier
		Conductible concurrently to other implementation projects	Facilitator
	Incentive systems	Financial incentive systems	Facilitator
	Mission alignment	Alignment with vision and goals of the general practice (eg, progressiveness/innovation, minimising medication usage, providing tailored patient care)	Facilitator
	Funding	Sufficient internal financial resources	Facilitator
	Materials and equipment	Most patients possess smartphone and earphones	Facilitator
		Prior possession of required equipment	Facilitator
	Access to knowledge and information	General practitioner not reached by information aimed at secondary healthcare	Barrier
		Providing educational materials to general practitioners	Facilitator
		Publications in accessible medical literature	Facilitator
4. Individuals domain roles subdomain	High-level leaders	General practice management is independent from secondary healthcare facilities	Facilitator
		Joint policy-making with co-owning general practitioners	Barrier
	Mid-level leaders	Flat hierarchy in general practice	Facilitator
	Opinion leaders	Identifiable and helpful	Facilitator
		Varies depending on implementation project subject	Barrier
	Implementation leads	Identifiable and helpful	Facilitator
		Varies depending on implementation project subject	Barrier
	Innovation recipients	Generally inclined to use music interventions	Facilitator
Characteristics	Opportunity	Heavy workload	Barrier
Subdomain		Understaffing or frequent changes in staff	Barrier

Key barriers and facilitators as perceived by the participants and categorised according to the updated CFIR.

**Figure 1:**
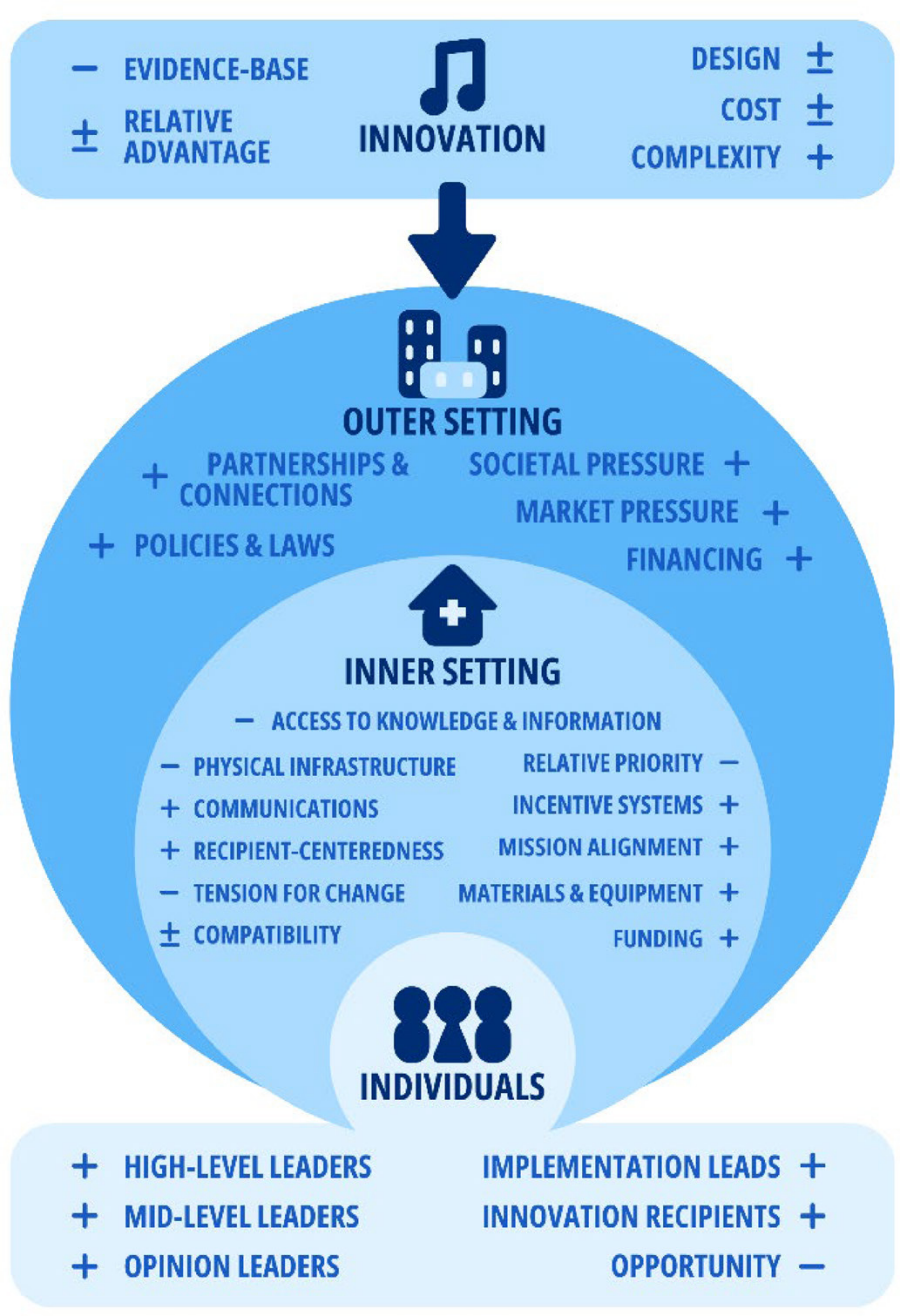
Constructs within the four examined domains of the Updated Consolidated Framework for Implementation Research, identified as key barriers and/or facilitators for implementation of perioperative music interventions in the general practice. − indicates a barrier. + indicates a facilitator. ± indicates a construct identified as both a barrier and facilitator for implementation.

A total of 19 barriers and 34 facilitators were identified (see figure 1 for a graphic presentation according to the CFIR constructs and online supplemental file 2 for the complete list of facilitators and barriers).

### Innovation domain

Regarding the innovation domain, GPs reported having insufficient prior knowledge of perioperative music interventions and the existing evidence-based research. Moreover, GPs noted a current lack of evidence-based research conducted in primary care. Participants concurred that research conducted in a hospital care setting could not be extrapolated to general practice, due to differences in surgical complexity and the associated pain and anxiety experienced by the patients. However, half of all participants denied this being an impediment to implementation of perioperative music interventions in their general practice.

[Most] of what you are doing as a general practitioner is not evidence-based. (participant 4)

GPs highlighted that delivery of music via headphones impaired communication with the patient, presenting as a crucial barrier for implementation. A speaker using wireless technology was proposed as an alternative mode of delivery. Using this mode of delivery, GPs revealed a preference for a maximum loudness over which conversation remains possible. Generally, GPs were receptive towards playing musical content of the patient’s preference, with the exception of some more extreme genres (eg, heavy metal, electronic dance music with very strong beats).

I actually quite enjoy all sorts of music. However, some sort of heavy metal-type music would be unworkable. (participant 11)

Even if [the musical content] is not my preference, I do not have to endure it for hours. I perform a procedure, but at most (…) it lasts maybe twenty to twenty-five minutes. (participant 3)

GPs concurred that music interventions could most feasibly be implemented intraoperatively, rather than preoperatively or postoperatively, due to constraints in available time and space. Some participants proposed the possibility of advising patients to listen to music at home.

Generally, the low expected costs of the required equipment turned out as a facilitator for implementation. However, some GPs raised concerns regarding costs associated with licences required for public utilisation of copyrighted music. The user-friendliness of perioperative music interventions was considered a crucial facilitator for implementation in primary care, lowering the threshold for wider-scale adoption of the intervention.

… it should be easy to use. That is actually—if it is not easy to use, it will just not be used at all. That is the experience in primary care. (participant 1)

Some GPs preferred non-pharmacological interventions to treat patients as opposed to pharmacological interventions whenever possible. Participants noted only very rarely prescribing benzodiazepines and opiates to treat perioperative anxiety and pain, whereas local anaesthesia was often sufficient. Thus, most GPs identified an additive role for perioperative music interventions as opposed to a substitute for existing treatment options.

### Outer setting domain

Participants mentioned affiliations with both regional and national collaborations, presenting as a facilitator for the implementation of music interventions. Participants highlighted that regional primary care coalitions are often closely involved with their members, provide educational information and conferences, and finance and facilitate innovation projects. Furthermore, national collaborations organise educational conferences and provide primary care clinical practice guidelines. Affiliations with academic networks appeared less common, as it seemed heavily dependent on the GP’s involvement in the supervision of GPs in training.

If you want to expand something, such as research or what have you, then you should approach a regional care collaborative. [They maintain] a solid foothold, since everybody has contact with them. And they implement more things similar to this. (participant 15)

Most participants highlighted that including perioperative music interventions in primary care clinical practice guidelines would facilitate the implementation process, resulting in increased acceptance on a broader scale. However, the current absence of perioperative music interventions in primary care guidelines did not constitute a barrier for implementation. Additionally, some GPs mentioned that the current inclusion of perioperative music interventions in hospital care guidelines presented as a facilitator for implementation in primary care, noting that inclusion in a hospital care guideline requires considerable effort and evidence and thus provides a steppingstone for implementation in general practice.

When I conceive of something myself, it does not matter as much. However, if it is included in a guideline, then I do think that it will be embraced to a greater extent in its entirety. (participant 10)

Although all participants denied experiencing negative peer pressure when deciding whether or not to initiate implementation projects, positive peer pressure presented as a facilitator for implementation by enabling inspiration of other GPs via word of mouth. Moreover, pressure exerted by patients to use music interventions in general practice was mentioned as a facilitator, with some participants indicating a potential role for patient organisations and promotion of the intervention via conventional and social media.

Access to external financing, such as subsidies or provision of required equipment, presented as a potential facilitator for implementation of music interventions. However, absence of possibilities for external financing did not constitute a barrier for implementation.

### Inner setting domain

Within the inner setting domain, the lack of readily accessible information aimed at primary care regarding perioperative music interventions constituted a barrier for implementation. Existing information aimed at hospital care physicians (eg, hospital care clinical practice guidelines) did not seem to adequately reach general practices. Participants highlighted the demand for information aimed at primary care physicians, promoting distribution via educational meetings and publication of research articles in more accessible, generalist medical literature.

As a general practitioner, you just do not know about all these [hospital care] guidelines. You can search for them, and you can find them, but there is just not enough time to review them all. (participant 4)

The interviews revealed some scepticism towards the utility of perioperative music interventions in general practice, presenting as a barrier for implementation. Treatment of the pain associated with minor surgical procedures performed in primary care was generally not perceived as a significant issue, and perioperative anxiety was addressed through communication with the patient. However, partial nail extractions and insertion of intrauterine devices were explicitly mentioned as potential procedures with the greatest added benefit for music interventions, due to the higher levels of associated pain and anxiety, respectively.

Despite GPs noting being involved in a multitude of implementation projects and therefore potentially not prioritising implementation of perioperative music interventions, most participants deemed it not very time-consuming and thus conductible concurrently with other implementation projects.

It [implementation of music interventions] requires little effort. So I think I could implement it immediately. At the next procedure even, so to speak. (participant 13)

Most GPs expressed having sufficient financial resources to implement perioperative music interventions. Additionally, some GPs mentioned already possessing the required materials, posing a facilitator for further implementation. Some participants emphasised the possibility of patients using their smartphone and earphones for music delivery. When using a speaker for music delivery, poor noise-insulating properties of general practice could constitute a barrier for implementation.

Generally, there was a shared belief among team members on minimising perioperative pain and anxiety, posing a facilitator for implementation of music interventions. A smaller team size and possibilities for frequent peer consultation and discussion constituted facilitators by enabling accessible and approachable communication between team members. Additionally, participants mentioned implementation of perioperative music interventions aligning with the vision and goals of their general practice, such as progressiveness and innovation, minimalising medication usage and providing patient-tailored care.

Some participants raised concerns regarding the incorporation of music interventions into their workflow, due to the often very limited available time per consultation. Conversely, GPs mentioned often diagnosing a condition prior to scheduling the surgical procedure for another consultation, presenting the option to extend the required efforts over multiple consultations. Moreover, potential benefits of music interventions regarding the concentration and overall performance of the operating physician were expressed as a potential facilitator for implementation.

Now, I inform the patients the moment they are lying anxiously on my examination table. I think that is too late. (…) Maybe it would be a better idea to just introduce it [music interventions] immediately when scheduling the appointment. (participant 3)

Generally, an absence of incentive systems did not constitute a barrier for implementation. However, if incentive systems should be applied, consensus was that a financial stimulus would be most facilitating for implementation of music interventions.

### Individuals domain

The interviews revealed that GPs experience a heavy workload, understaffing and frequent staff changes, presenting as barriers for implementation.

Additionally, several key facilitators were identified. GPs mentioned having an autonomous role regarding the management of their general practice, independent of secondary healthcare facilities and other general practices. Although frequently accompanied by co-owning GPs with whom a joint general practice policy is coordinated, only some GPs noted this as a potential barrier for implementation.

We are of course rather independent. We do not operate within a hospital structure. So if we want to try something, and we are up for it—I could implement it [music interventions] on my own, or I could include my colleagues. We are significantly more autonomous than [physicians] in a hospital (participant 8)

Physicians employed as salaried GPs, GPs in training or locum GPs, were often not directly involved in policy-making. However, this did not constitute a barrier for implementation according to both physicians of these employment types as general practice owners. Due to the flat hierarchical structure experienced in general practice, the setting is receptive towards suggestions made by all physician types. Furthermore, most GPs expected their patients to largely accept music interventions as part of their perioperative care.

I think so. Most of them would [accept music interventions]. And I do not think many patients would decline. (…) I do not think that it would be an issue. (participant 9)

Lastly, most GPs expressed identifying individuals that could facilitate implementation by leading the implementation process (ie, implementation champions) or informally influencing attitudes and behaviour of others (opinion leaders). However, these individuals could vary depending on the implementation project.

### Secondary outcomes

Of the 15 participants, 11 (73.3%) were willing to implement perioperative music interventions in their general practice. A demand for tailored provision of information, the heavy workload of daily practice and being involved in other implementation projects were cited as reasons among those not willing to implement. Four (26.7%) participants believed that perioperative music interventions should be part of standard perioperative care. Cited reasons for not incorporating music interventions into standard perioperative care were: a demand for evidence-based research in primary care, initially preferring a small-scale trial and allowing other GPs to decide autonomously whether or not to implement perioperative music interventions.

## Discussion

This study examined the barriers and facilitators for implementation of music interventions in general practice. The hypothesis was that these would be different from the barriers and facilitators identified in a hospital care setting.^18^ As in hospital care, a lack of knowledge of the effect of music interventions is a key barrier for implementation. Unlike hospital care professionals, GPs signal that there is limited research performed in their setting into the effectiveness of music interventions. Another key difference is that GPs view their working environment as a facilitator for implementation of perioperative music interventions. The autonomy of GPs and the smaller-scale teams working in primary care are seen as enabling factors for implementation in contrast to larger and varying teams in hospital care.

This study highlights that information regarding perioperative music interventions does not adequately reach general practice. This was supported by participants expressing having insufficient prior knowledge to the point of total unawareness of the concept of perioperative music interventions. In a systematic review investigating the adoption of clinical practice guidelines in primary care, a lack of knowledge was also identified as a significant barrier for implementation.^27^ Alternatively, developing knowledge of guideline content through provision of education and training appeared a facilitator,^27^ supporting the demand among GPs emphasised in this study for targeted provision of educational materials and publications in accessible medical literature.

Within the ‘innovation domain’ of CFIR, insufficient prior knowledge of perioperative music interventions and lack of research conducted in primary care were identified as key barriers by half of the participants. This implies that GPs may be inclined to implement perioperative music interventions regardless of (their knowledge of) the existing evidence-base. A cross-sectional survey among Dutch GPs revealed that two-thirds of GPs witnessed low-value care (ie, care with minimal to no benefit, considering the harms, costs and alternatives^28^ being provided regularly or often within general practice, most frequently with the aim to uphold a good physician–patient relationship).^29^ Therefore, even if knowledge of the evidence-base is insufficient or its applicability to primary care is considered inadequate, GPs may still be inclined to implement perioperative music interventions if they believe it to be beneficial to this purpose.

Although headphones have become the predominant method of delivery for perioperative music interventions in the hospital care setting,^10 12^ this study reveals that GPs consider delivery of music via headphones a crucial barrier for implementation, as it impedes communication between physician and patient. Empathic, supportive and patient-centred communication have been associated with improved health outcomes in general practice patients.^30^ Therefore, intervention designs that impede physician-patient communication—although due to delivery of music via headphones, excessive loudness of the music or more extreme musical content (eg, heavy metal)—may experience reduced uptake among GPs. A loudspeaker was suggested as a potential alternative mode of music delivery in this study.

Moreover, this study reveals user-friendliness of perioperative music interventions to be a crucial facilitator for implementation in general practice. Since Dutch GPs increasingly suffer from a heavy workload and understaffing,^27 31^ interventions requiring excessive time and effort will probably not be prioritised for implementation and will not be used in practice. Insufficient time for reflecting on and evaluating the implementation process also presented as a barrier in the IMPROVE study in hospital care.^18^ Furthermore, this study reveals that the limited available time per consultation (10–15 min for a regular consultation; approximately 20 min for surgical procedures) impedes incorporation of perioperative music interventions into the workflow of GPs. While the hospital care setting offers opportunities for providing information and equipment to the patient in nursing wards or the preoperative holding area,^9 13 14^ GPs are required to fit in all aspects of delivering the intervention in a shorter timeframe, due to the often autonomous and solitary way of providing care to patients with a high turnover rate. A national survey investigating contextual factors regarding the use of non-drug interventions among Australian GPs supports this finding, stating that half of the participants experienced insufficient time to address non-drug interventions during consultation with the patient.^32^ Therefore, the selection of equipment, provision of information to patients, installation and operation of the intervention should require minimal effort to lower the threshold for widespread adoption.

Additionally, inclusion of perioperative music interventions in primary care practice guidelines facilitates the implementation process. Generally, GPs have a positive attitude towards utilisation of guidelines.^33^ Since GPs find it difficult to deviate from guidelines based on past experiences or patient preferences^34 35^—guidelines which currently do not include perioperative music interventions—uptake may be enhanced if perioperative music interventions were included in primary care guidelines. Although inclusion in hospital care guidelines did also present as a facilitator for some GPs in this study, it does not substitute guidelines targeted at primary care. The absence of evidence conducted in primary care and lack of access to hospital care guidelines warrant a targeted consideration of the evidence to construct guidelines specific to general practice situation.

Additionally, this study highlights a reduced expected utility of perioperative music interventions in general practice. Perioperative anxiety and pain are perceived as less significant issues and the standard treatment, involving communication with the patient, local anaesthesia and an occasional non-opioid analgesic, generally suffices. In hospital care settings, the prevalence of perioperative anxiety has been reported as high as 72% of cases.^36^ Although no literature is available regarding the epidemiology of perioperative anxiety and pain in general practice, anxiety and pain have been established in patients undergoing procedures with injections^37^ (such as venipuncture or local infiltration anaesthesia), women considering or undergoing the insertion of intrauterine devices^38 39^ and patients undergoing surgical treatment of ingrown toenails.^40^ Considering that these procedures are closely related to those performed in the Dutch general practice, this raises the question whether surgery-related anxiety, stress and pain are actually less prevalent in general practice situation, or rather underestimated by GPs. A meta-analysis by Ruben *et al* has shown that physicians significantly underestimate the pain associated with (surgical) procedures experienced by patients.^41^ Moreover, promising results of perioperative music interventions have already been shown in some of the settings, namely venipuncture and local infiltration anaesthesia.^42 43^ Therefore, the utility of perioperative music interventions in general practice may be greater than expected.

Lastly, this study reveals a high willingness among GPs to implement perioperative music interventions in general practice, with 73.3% of participants willing to implement. In the hospital care setting, a similar rate of 80% was identified after implementation efforts had been made.^9^

### Limitations

This study has several limitations. First, volunteer bias may have been introduced. GPs most intrigued by the implementation of perioperative music interventions may have been more inclined to participate in this study, which is supported by the finding that 40% of participants had previously used perioperative music interventions. This may have resulted in an overestimation of (the importance of) the facilitators for implementation and the willingness to implement perioperative music interventions, and an understatement of the barriers for implementation. Second, the process of data collection and analysis was conducted by a single investigator. Interviewer bias may have influenced the participants’ responses and thus biased the identified barriers and facilitators. Moreover, identification of codes from the qualitative data and subsequent classification within the constructs and domains of the Updated CFIR requires a certain degree of interpretation and thus introduces potential inter-investigator variability. Lastly, despite achieving data-saturation by including 15 participants, the limited sample size may reduce generalisability of the study results to a broader context. The extensive overview of barriers and facilitators notwithstanding, the perceived importance of specific contextual factors may differ if the study results were to be validated among a larger study population. Theoretically, the different recruitment strategies to find participants might have led to different types of respondents (Rotterdam area vs The Netherlands). However, we observed no differences in responses stratified by recruitment strategy which makes it unlikely that bias was introduced that way.

### Strengths

To our knowledge, this study is the first insight into implementation and utilisation of perioperative music interventions in general practice situations. Due to its distinction from hospital care, general practice requires a targeted assessment of contextual factors to ensure implementation of perioperative music interventions with durable and sustainable results. Moreover, the composite approach for recruitment of participants has resulted in the inclusion of GPs from varying positions within the hierarchical structure, and from independent general practice situations. Furthermore, utilisation of the CFIR framework enabled comprehensive assessment of contextual factors and classification according to one of the most frequently cited frameworks in implementation science.^20 44^

### Implications of the findings

This study provides tools to guide future research and implementation efforts of perioperative music interventions in general practice situations. External financing, media pressure and inclusion of perioperative music interventions in primary care clinical practice guidelines may facilitate uptake of the intervention. Future research should address the epidemiology of perioperative anxiety, stress and pain in general practice to ascertain the added benefit of perioperative music interventions in this context. Additionally, future research should address the efficacy of perioperative music interventions in the treatment of these symptoms in general practice situations and the identification of effective implementation strategies to further guide implementation efforts with durable and sustainable results.

## Conclusions

Barriers and facilitators for the implementation of perioperative music interventions in general practice were identified among GPs and GPs in training. Some barriers and facilitators identified in general practice were not previously identified in a hospital care setting. Key barriers for implementation included insufficient prior knowledge, a lack of research conducted in primary care, impaired communication between physician and patient due to delivery of music via headphones or excessive loudness and a heavy workload. Key facilitators included user-friendliness, equipment costs and possibilities for external financing, affiliations with regional and national collaborations, inclusion in both primary and secondary clinical practice guidelines, the autonomous position of general practice owners and a flat hierarchical structure. Willingness among GPs to implement perioperative music interventions was high (73.3%).

## Supplementary material

10.1136/bmjopen-2025-114312 online supplemental file 1

## Data Availability

All data relevant to the study are included in the article or uploaded as supplementary information.
