# Supplementary material for: What are barriers and facilitators for implementation of music interventions for minor surgical procedures in general practice in the Netherlands? A qualitative study
Source: BMJ Open. 2026 Jul 15;16(7):e114312. doi: 10.1136/bmjopen-2025-114312 (PMC13374461; doi:10.1136/bmjopen-2025-114312)
Supplement: online supplemental file 1 [file bmjopen-16-7-s001.docx]

# Supplementary materials

## Supplementary File 1.

## The interview format developed and utilized in this study

### 1. General questions

1. What is your type of employment?
   (i.e. general practice owner, salaried general practitioner, locum general practitioner, general practitioner in training, or other)
2. How many physicians, including general practitioners in training, are employed at your general practice?
3. How long have you been actively employed as a general practitioner (in training)?
4. What is your year of birth?
5. What is your sex?
6. Have you heard about the effects of perioperative music interventions or other music interventions in the general practice prior to this study?
7. Have you priorly utilized music in the general practice?
8. In what manner have you utilized music interventions in the general practice? (e.g. in the waiting room, perioperatively, during clinical examination of patients, consultations with specific patient groups)
   With what purpose?

### 2. Barriers and facilitators

#### Innovation domain

1. What do you know of the effectiveness of perioperative music interventions?
2. Do you think the effect of perioperative music interventions has been adequately researched? If not, what research is missing?
3. What would be the ideal design of perioperative music interventions in your general practice, and why? (e.g. mode of delivery, timing of the intervention, musical content)
4. What influence do you think perioperative music interventions may have on the communication between you and the patient?
5. What influence do you think perioperative music interventions may have on your own functioning during minor surgical procedures?
6. How often do you prescribe pharmacologic agents, such as benzodiazepines and non-steroidal anti-inflammatory drugs/opioids, in the context of minor surgical procedures? Are you worried about the medication usage in the context of perioperative anxiety and pain?
7. Do you think perioperative music interventions could pose a non-pharmacological alternative in the treatment of perioperative anxiety and pain?
8. Are there any other characteristics of the intervention which could pose barriers or facilitators for implementation? (e.g. cost, complexity, usability, et cetera)

#### Outer setting domain

1. Do you think partnerships and connections exist that could facilitate implementation of perioperative music interventions? (e.g. networks, academic affiliations, professional associations, conferences)
   If not, what partnerships and connections should be established?
2. Do you think implementation of perioperative music interventions should be supported by clinical practice guidelines?
3. At this moment, perioperative music interventions are not included in Dutch primary care clinical practice guidelines. However, perioperative music interventions are included a secondary care guideline by the Dutch Society of Anesthesiologists and the Dutch Society for Surgery. This guideline recommends considering the application of perioperative music interventions in clinical surgical patients. Does this pose a barrier of facilitator for implementation of perioperative music interventions in the general practice?
4. Do you think that external financing is necessary for implementation of perioperative music interventions? (e.g. subsidies, grants)
   If yes, how should external financing be arranged?
5. Would you experience peer pressure to implement perioperative music interventions? Do you experience peer pressure at this moment?
6. Are there any other external influences that could pose a barrier or facilitator for implementation of perioperative music interventions? (e.g. governmental influences, laws and policies, environmental factors, sociocultural factors, media influence)

#### Inner setting domain

1. Do you have access to the required financial means to implement perioperative music interventions?
2. Do you have access to the required materials and equipment to implement perioperative music intervention with the design of your preference?
3. Researchers often focus on perioperative music interventions using headphones to deliver the music to the patient. Do you have access to the required materials and equipment to implement perioperative music interventions using this method?
4. Are you satisfied with the communication and teamwork at your general practice? Does this pose a barrier or facilitator for implementation of perioperative music interventions?
5. Is there a shared belief regarding the care for (needs of) patients amongst the physicians at your general practice? How does this influence the implementation of perioperative music interventions?
6. Does your general practice provide a safe environment for working and learning? How does this influence the implementation of perioperative music interventions?
7. How do positive incentives (rewards) or negative incentives (punishments) influence implementation of perioperative music interventions? What should these positive and/or negative incentives be?
8. Does implementation of perioperative music interventions align with the vision or goals of the general practice as an organization? (e.g. innovative or progressive care)
   Does this pose a barrier or facilitator for implementation of perioperative music interventions?
9. Is your general practice involved in other implementation projects with the aim to reduce perioperative anxiety and pain? And implementation projects other than the aforementioned? Does this pose a barrier or facilitator for the implementation of perioperative music interventions?
10. Are there any other contextual factors within the general practice that could pose a barrier or facilitator for implementation of perioperative music interventions? (e.g. physical space, technological factors, knowledge and skills, et cetera)

#### Individuals domain

1. Who decides policy-making at your general practice, such as deciding to implement perioperative music interventions? Does this pose a barrier or facilitator for implementation of perioperative music interventions?
2. Are there identifiable opinion leaders employed at your general practice? (i.e. individuals with informal influence on the attitudes and behavior of others)
   Does this pose a barrier or facilitator for implementation of perioperative music interventions?
3. Are there identifiable implementation champions employed at your general practice? (i.e. individuals who lead efforts to implement innovations)
   Does this pose a barrier or facilitator for implementation of perioperative music interventions?
4. Do you think the patients would be inclined to utilize perioperative music interventions? What factor would impede them to use perioperative music interventions? What factors would facilitate this?
5. Are there any other contextual factors regarding the individuals that could pose a barrier or facilitator for implementation of perioperative music interventions? (e.g. authority/hierarchy, individual needs, motivation, availability)

### 3. Secondary outcomes

1. Are you willing to implement perioperative music interventions in your general practice?
2. Do you think music interventions should be part of standard perioperative care in the general practice?

## Supplementary File 2. All barriers, facilitators and neutral factors as perceived by the participants and categorized according to the Updated Consolidated Framework for Implementation Research (CFIR)

**CFIR domain Construct Codes Determinant**

**1. Innovation domain** Source Tendency for general practitioners to be critical of the Barrier
 information source

Evidence-Base Insufficient prior knowledge Barrier

Lack of research conducted in primary healthcare Barrier

Relative Advantage Preference for nonpharmacological interventions Facilitator

Standard treatment rarely includes additional medication Barrier
prescriptions, is effective and/or irreplaceable

Lack of research comparing music interventions to standard Barrier
treatment

Allows minor surgical procedures for patients otherwise too Facilitator
anxious

Adaptability Music intensity and content adaptable to patient preferences Facilitator

Mode of delivery adaptable (i.e. speaker versus headphones) Facilitator

Trialability Testable with a few patients before scaling up Facilitator

Testable with smartphone before purchasing more appropriate Facilitator
equipment

Complexity User-friendly & not difficult to install Facilitator

Establishing Bluetooth/Wi-Fi connection Barrier

Errors & troubleshooting Barrier

Design Recognizable product (e.g. with hospital logo) Facilitator

Communication impaired by headphones Barrier

Open attitude of general practitioners towards musical content Facilitator
of the patient’s preference, except extreme genres

Inability of patients to express musical content preference Barrier

Premade music playlists Facilitator

Communication impaired by music loudness Barrier

Ability of patient to connect and set up the intervention Facilitator

**CFIR domain Construct Codes Determinant**

**1. Innovation domain** Design Patients receptive for lifestyle advice, such as listening to music Facilitator

Cleanliness of the mode of delivery Barrier

Cost Affordable equipment Facilitator

License to utilize copyrighted music Barrier

**2. Outer setting** Critical Incidents COVID-19 restrictions Barrier

**domain**  Local Attitudes Cultural differences in attitude towards music Neutral

Reluctancy amongst older patients Barrier

Familial hierarchy of the patient Neutral

Local Conditions Low socioeconomic status of the patient Barrier

Partnerships & Connections Local general practitioner groups provide inspiration and peer Facilitator
consultation

Regional collaboratives provide reach, education, funding, Facilitator
 facilitation and contact with health insurance providers

National collaboratives provide reach, education, guidelines Facilitator
and represent interests of general practitioners

Academic centers provide reach and education Facilitator

General practitioner emergency posts provide reach Facilitator

Policies & Laws Existing guideline aimed at secondary healthcare Facilitator

Development of guideline aimed at general practitioners Facilitator

Guidelines overly restrictive Barrier

Privacy legislation concerns Barrier

Financing External financing (e.g. equipment, financial compensation) Facilitator

Limitation of financing to initial implementation phase Barrier

Financial arrangements with music performance rights Facilitator organizations

External Pressure - -

Societal Pressure Pressure exerted by patients and patient organizations Facilitator

Media pressure (e.g. conventional media, social media) Facilitator

Market Pressure Positive peer pressure provides inspiration Facilitator

Indifference amongst general practitioners towards negative Barrier
peer pressure

**CFIR domain Construct Codes Determinant**

**2. Outer setting** Performance-Measurement Accreditation by national collaboratives Facilitator
**domain** Pressure

**3. Inner setting** Structural Characteristics - -

**domain** Physical Infrastructure Separate operating room present in general practice Facilitator

Inadequate noise-insulating properties Barrier

Information Technology Need for subscription to music provider service Barrier
Infrastructure

Work Infrastructure - -

Relational Connections Approachable, familiar and long-term physician-patient Facilitator
relationship

Communications Satisfaction with quality of team work and communication Facilitator

Possibilities for frequent peer consultation and discussion Facilitator

Short lines of communication due to smaller team size Facilitator

Motivating communications between physician and patient Facilitator
 increase uptake and adherence

Culture - -

Human Equality-Centeredness - -

Recipient-Centeredness Shared belief on minimizing patient anxiety and pain Facilitator

Deliverer-Centeredness Caring for welfare of colleagues improves patient care Facilitator

Learning-Centeredness Safe learning environment Facilitator

Tension for Change Reduced patient anxiety and pain due to minor, brief and less Barrier
 complex surgical procedures

Reduced patient anxiety due to communication with physician Barrier

Higher levels of anxiety and pain in patients undergoing Facilitator
insertion of intrauterine devices and partial nail extractions
respectively

Compatibility Improves concentration and functioning of operating physician Facilitator

Limited time per consultation for minor surgical procedures Barrier

Diagnosing consult and separate scheduled surgical consult Facilitator

Clustered consultations specifically for surgical procedures Facilitator

Not part of routine perioperative care Barrier

Relative Priority Involvement in multitude of implementation projects Barrier

**CFIR domain Construct Codes Determinant**

**3. Inner setting** Relative Priority Conductible concurrently to other implementation projects Facilitator

**domain** Incentive Systems Financial incentive systems Facilitator

Competitiveness Facilitator

Mission Alignment Alignment with vision and goals of the general practice Facilitator

(e.g. progressiveness/innovation, minimalizing medication
usage, providing tailored patient care)

Available Resources - -

Funding Sufficient internal financial resources Facilitator

Expenses shared between co-owning general practitioners Facilitator

Space - -

Materials & Equipment Most patients possess smartphone and earphones Facilitator

Prior possession of required equipment Facilitator

Misplacement of portable equipment Barrier

Purchasing the most appropriate equipment Barrier

Access to Knowledge & Information General practitioner not reached by information aimed at Barrier
 secondary healthcare

Providing educational materials to general practitioners Facilitator

Publications in accessible medical literature Facilitator

Providing (digital) educational materials to patients Facilitator

**4. Individuals domain** High-level Leaders General practice management is independent from secondary Facilitator
**Roles subdomain** healthcare facilities

Autonomous position of general practice owners Facilitator

Joint policy-making with co-owning general practitioners Barrier

Mid-level Leaders Flat hierarchy in general practice Facilitator

Opinion Leaders Identifiable and helpful Facilitator

Varies depending on implementation project subject Barrier

Implementation Facilitators Assistance from technology proficient individuals Facilitator

Implementation Leads Identifiable and helpful Facilitator

Varies depending on implementation project subject Barrier

Implementation Team Members Support from doctor’s assistants Facilitator

Other Implementation Support - -

**CFIR domain Construct Codes Determinant**

**4. Individuals domain** Innovation Deliverers Industry supports providing of equipment Facilitator

**Roles subdomain** Innovation Recipients Generally inclined to utilize music interventions Facilitator

Innovation Recipients Not familiar with/negative attitude towards music interventions Barrier

Overstatement of the impact of minor surgical procedures due Barrier
 to introduction of an additional intervention

**Characteristics**  Need - -

**subdomain** Capability Competent innovation delivering physicians Facilitator

Difficult for computer illiterate employees Barrier

Opportunity Heavy workload Barrier

Understaffing or frequent changes in staff Barrier

Motivation Motivated intervention delivering physician Facilitator

Supplementary File 3: COREQ checklist

Consolidated criteria for reporting qualitative studies (COREQ): 32-item checklist

Developed from:

Tong A, Sainsbury P, Craig J. Consolidated criteria for reporting qualitative research (COREQ): a 32-item checklist for interviews and focus groups. International Journal for Quality in Health Care. 2007. Volume 19, Number 6: pp. 349 – 357

| **Item No** | | **Guide Questions/Description** | **Answer/reported on page #** |  |
| --- | --- | --- | --- | --- |
| **Domain 1: Research team and reflexivity** | | | |  |
| **Personal Characteristics** | | | |  |
| 1. Interviewer/ facilitator | | Which author/s conducted the interview or focus group? | MvE |  |
| 2. Credentials | | What were the researcher’s credentials? E.g., PhD, MD | BSc. |  |
| 3. Occupation | | What was their occupation at the time of the study? | Medical student |  |
| 4. Gender | | Was the researcher male or female? | Male |  |
| 5. Experience and training | | What experience or training did the researcher have? | Medical research curriculum |  |
| **Relationship with participants** | | | |  |
| 6. Relationship established | | Was a relationship established prior to study commencement? | No |  |
| 7. Participant knowledge of the interviewer | | What did the participants know about the researcher? e.g. personal goals, reasons for doing the research? | Pg 5, no personal information except name and occupation |  |
| 8. Interviewer characteristics | | What characteristics were reported about the interviewer/facilitator? e.g. Bias, assumptions, reasons and interests in the research topic | N.A. |  |
| **Domain 2: study design** | | |  |  |
| **Theoretical framework** | | |  |  |
| 9. Methodological orientation and Theory | What methodological orientation was stated to underpin the study? e.g. grounded theory, discourse analysis, ethnography, phenomenology, content analysis | Pg 5-7 |  |  |
| **Participant selection** | | |  |  |
| 10. Sampling | How were participants selected? e.g., purposive, convenience, consecutive, snowball | Pg 5-6 |  |  |
| 11. Method of approach | How were participants approached? e.g., face-to-face, telephone, mail, email | Pg 5 |  |  |
| 12. Sample size | How many participants were in the study? | 15 |  |  |
| 13. Non-participation Setting | How many people refused to participate or dropped out? Reasons? | Unknown, given the use of (social) media for recruitment |  |  |
| 14. Setting of data collection | Where was the data collected? e.g., home, clinic, workplace | Pg 5-7; in person interviews conducted at varying locations, mostly the participant’s workplace |  |  |
| 15. Presence of nonparticipants | Was anyone else present besides the participants and researchers? | No |  |  |
| 16. Description of sample | What are the important characteristics of the sample? e.g. demographic data, date | Pg 9-10 |  |  |
| **Data collection** | | |  | No |
| 17. Interview guide | Were questions, prompts, and guides provided by the authors? Was it pilot tested? | Pg 7, interview format available in supplementary data. Not pilot tested, but 4 iterations of revision by research team |  |  |
| 18. Repeat interviews | Were repeat interviews carried out? If yes, how many? | No |  |  |
| 19. Audio/visual recording | Did the research use audio or visual recording to collect the data? | Audio |  |  |
| 20. Field notes | Were field notes made during and/or after the interview or focus group? | No |  |  |
| 21. Duration | What was the duration of the interviews or focus group? | 45-70 minutes |  |  |
| 22. Data saturation | Was data saturation discussed? | Pg 23 |  |  |
| 23. Transcripts returned | Were transcripts returned to participants for comment and/or correction? | No |  |  |
| **Domain 3: analysis and findings** | | |  |  |
| **Data analysis** | | |  |  |
| 24. Number of data coders | How many data coders coded the data? | 1 |  |  |
| 25. Description of the coding tree | Did the authors provide a description of the coding tree? | No |  |  |
| 26. Derivation of themes | Were themes identified in advance or derived from the data? | Themes identified in advance, coding derived of the data |  |  |
| 27. Software | What software, if applicable, was used to manage the data? | Nvivo, version 15 |  |  |
| 28. Participant checking | Did participants provide feedback on the findings? | No |  |  |
| **Reporting** | | |  |  |
| 29. Quotations presented | Were participant quotations presented to illustrate the themes/findings? Was each quotation identified? e.g., participant number | Yes |  |  |
| 30. Data and findings consistent | Was there consistency between the data presented and the findings? | Yes, pg 19-23 |  |  |
| 31. Clarity of major themes | Were major themes clearly presented in the findings? | 9-18 |  |  |
| 32. Clarity of minor themes | Is there a description of diverse cases or a discussion of minor themes? | 9-18 |  |  |
