# Supplementary material for: What are barriers and facilitators for implementation of music interventions for minor surgical procedures in general practice in the Netherlands? A qualitative study
Source: BMJ Open. 2026 Jul 15;16(7):e114312. doi: 10.1136/bmjopen-2025-114312 (PMC13374461; doi:10.1136/bmjopen-2025-114312)
Supplement: Supplementary file 2 [file bmjopen-2025-114312.reviewer_comments.pdf]

PEER REVIEW HISTORY

BMJ Open publishes all reviews undertaken for accepted manuscripts. Reviewers are asked to complete a checklist review form and are provided with free text boxes to elaborate on their assessment. These free text comments are reproduced below.

This paper was submitted to another journal from BMJ but declined for publication following peer review. The authors addressed the reviewers' comments and submitted the revised paper to BMJ Open. The paper was subsequently accepted for publication at BMJ Open.

ARTICLE DETAILS

Title (Provisional)

What are barriers and facilitators for implementation of music interventions in general practice situations in The Netherlands? – A qualitative study

Authors

van Engelen, Milan; Verhoeven, Jorrit G.; Ista, Erwin; Schiphof, Dieuwke; Jeekel, Johannes; Klimek, Markus

---

VERSION 1 - REVIEW

**Reviewer**	**1**
**Name**	**Reviewer 1**
**Affiliation**	
**Date**	**18-Nov-2025**
**COI**	**No competing interest**

Comments not published pending reviewer's consent

VERSION 2 - REVIEW

**Reviewer**	**1**
**Name**	**Westlake, Debra**
**Affiliation**	**University of Oxford, Nuffield Department of Primary Care Health Sciences**
**Date**	**04-Feb-2026**
**COI**	**None**

Review of article

This represents an interesting article about the barriers and facilitators to the use of perioperative music by general practitioners in the Netherlands when performing minor surgery in their practice setting. I believe the authors are qualified or trainee doctors in general practice, anaesthesiology, neurology and other connected specialisms. A comparison is made to the use of music in hospital settings for similar purposes and one study in particular that applies an implementation framework (CFIR) to the research data collection and analysis. This framework is applied to this study. The study highlights the barriers and facilitators to using music perioperatively in general practice in the Netherlands.

Minor revisions

1. There are minor corrections in grammar and use of English needed throughout, although generally the standard is very high. The use of the definite article 'the' with 'general practice' is incorrect in normal usage in English and needs to be corrected throughout. As also in the use of 'priorly' when the authors mean previously.

Major revisions

2. While this is an exploratory study, there should still have been a research question which is stated at the beginning of the methods section so that we can determine if this is the most appropriate method for use to answer the question and how novel the study is. The field of investigation of the research seems to be on implementation science, but this is not made clear from the introduction.
3. For readers not familiar with the methods, themes and topics in the article these need much clearer explanation, particularly in the introduction which makes a number of claims without exploring them or explaining how ideas are linked. This is shown in the first and second sentence of the introduction where the authors claim that evidence that anxiety and stress and pain may be reduced by music interventions 'consequently' may mean they reduce the need for postoperative analgesia – without a clear causal explanation or full exploration of the mechanisms by which music may produce benefits to patients and clinicians.
4. As readers we need to understand something about theories of pain and the use of interventions like music – these are absent. We also do not at any point read references to patient experience of music and how patients have said it might help – without these the idea of implementation of such an intervention seems spurious. Also the generic use of the concept of 'music' is unhelpful as an intervention. As interviewees in quotations rightly point out, it depends on what type of music, who chooses it and how it is delivered and at what 'dose'. These must surely be factors in determining what the intervention actually is, before its

implementation can be devised and if it can have the desired outcomes? A clear logic model or theory of change would support the implementation study.

5. Page 9 the CFIR is broken down into elements but these are not explained in any detail – it would be helpful to have a box with the blank framework and brief key definitions of what 'inner setting' and 'outer setting ' mean for example. Not all readers will be familiar with theories of implementation science or this particular one.
6. Analysis: The description of analysis is unclear – what does bottom-up from the full qual text mean? I also wonder what about the analysis was inductive? Though the authors say it was inductive-deductive – this is not at all clear. It feels very much that analysis was weighted towards a deductive analysis and the CFIR framework was imposed on the data with little of the conceptual or deeper thought usually characteristic of qualitative analysis.

**Reviewer**	**2**
**Name**	**Chan, Sze Ling**
**Affiliation**	**Singhealth, Health Services Research Centre**
**Date**	**14-Apr-2026**
**COI**	**None**

---

1. Title submitted and title in manuscript are different

2. Introduction – more background into the types of procedures or patients where music interventions have been shown to be effective would be helpful. Is there evidence for minor procedures too? Also a brief description of music interventions would be helpful for those not familiar with it (e.g. is it just playing some music in the background, are there certain types of music used or can patients choose, etc).

3. Methods (study population) – there seems to be different recruitment strategies for greater Rotterdam and the rest of Netherlands. Was there a bias in the response based on region and would this have any implication on the results (i.e. do you expect differences in barriers and facilitators by region)?

4. Page 9 lines 32-37 (last sentence of data collection section) – this relates to the process domain of CFIR, which was excluded. It's also not in the interview guide. Pls clarify if this was part of the interview and the purpose of this since the process domain was excluded.

5. Supplementary table 1 can be in the main text

6. There's no supplementary file 3 but there's a file 4. Pls double check numbering.

VERSION 2 - AUTHOR RESPONSE

Reviewer 1:

Minor revisions

1. There are minor corrections in grammar and use of English needed throughout, although generally the standard is very high. The use of the definite article 'the' with 'general practice' is incorrect in normal usage in English and needs to be corrected throughout. As also in the use of 'priorly' when the authors mean previously.

Response: We thank the reviewer for the suggested corrections and have made these in the manuscript.

Major revisions

2. While this is an exploratory study, there should still have been a research question which is stated at the beginning of the methods section so that we can determine if this is the most appropriate method for use to answer the question and how novel the study is. The field of investigation of the research seems to be on implementation science, but this is not made clear from the introduction.

Response: We have incorporated a research question at the start of the methods section as suggested. We appreciate that the introduction has a rather long 'run-up' until it introduces the implementation science subject. The additional background information requested by the reviewers has not necessarily shortened this. We have therefore explicitly mentioned implementation in the research question in the abstract as well. We believe that it should now be sufficiently identifiable as an implementation study for readers.

3. For readers not familiar with the methods, themes and topics in the article these need much clearer explanation, particularly in the introduction which makes a number of claims without exploring them or explaining how ideas are linked. This is shown in the first and second sentence of the introduction where the authors claim that evidence that anxiety and stress and pain may be reduced by music interventions 'consequently' may mean they reduce the need for postoperative analgesia – without a clear causal explanation or full exploration of the mechanisms by which music may produce benefits to patients and clinicians.

Response: Reviewer 2 has made a similar suggestion and we have therefore expanded the introduction with more explanation of music interventions and the theory behind them. This includes references to review articles that do a great job at explaining the proposed mechanisms by which music produces benefits in detail.

Music has been used for millennia to achieve certain clinical benefits. Plato believed music could heal the soul, and Bach composed the Goldberg Variations to alleviate a Russian ambassador's insomnia. Dr. Evan O'Neil Kane was the first physician to use recorded music played through a phonograph in the operation theatre to ameliorate patients' anxiety in 1907.1 Modern science has found empiric evidence for these 'music interventions'. The analgesic effect of music interventions has been studied very well and a number of possible causal explanations have been put forward 2. Music has been suggested to function as a distractor from the pain experience. Another explanation is that music can elicit emotions that alleviate the pain experience.3 Functional MRI-scans have shown that listening to favorite music while undergoing a painful stimulus activates pain modulating areas of the brain 4.

In clinical research, a music intervention is often a defined 'medicine' with features set by the researchers. For example, in a study to investigate whether music interventions could reduce the incidence of delirium in patients undergoing brain surgery they were instructed to listen to their preferred music using headphones for 30 minute periods, before, and after surgery, and during the procedure itself .5 Clinical studies have shown that music interventions are effective in reducing the anxiety, stress and pain associated with surgical procedures in both adults and children and for a variety of procedures.6-11 Furthermore, perioperative music interventions may reduce the need for postoperative analgesia and increase patient satisfaction.8 10 12

4. As readers we need to understand something about theories of pain and the use of interventions like music – these are absent. We also do not at any point read references to patient experience of music and how patients have said it might help – without these the idea of implementation of such an intervention seems spurious. Also the generic use of the concept of 'music' is unhelpful as an intervention. As interviewees in quotations rightly point out, it depends on what type of music, who chooses it and how it is delivered and at what 'dose'. These must surely be factors in determining what the intervention actually is, before its implementation can be devised and if it can have the desired outcomes? A clear logic model or theory of change would support the implementation study.

Response: We believe this point also draws on point 3 made about the need for more explanation on the mechanism of music interventions

and we refer to that answer.

5. Page 9 the CFIR is broken down into elements but these are not explained in any detail – it would be helpful to have a box with the blank framework and brief key definitions of what 'inner setting' and 'outer setting ' mean for example. Not all readers will be familiar with theories of implementation science or this particular one.

Response: we have added a blank framework with definitions drawing directly from the CFIR-literature as a table:

Table 1: the CFIR-framework [24]

CFIR-framework*	Definition	Examples
1: Innovation Domain	*The "thing" being implemented*	*A new clinical treatment, educational program, or city service*
2: Outer Setting Domain	*The setting in which the Inner Setting exists,*	*Hospital system, school district, state. There may be multiple Outer Settings and/or multiple levels within the Outer Setting (e.g., community, system, state).*
3. Inner Setting Domain	*The setting in which the innovation is implemented,*	*Hospital, school, city. There may be multiple Inner Settings and/or multiple levels within the Inner Setting, e.g., unit, classroom, team.*
Individuals Domain	*The roles and characteristics of individuals.*	*Policy makers, managers, senior staff, junior staff, innovation recipients*

*The fifth domain; Implementation Process Domain was not under investigation in this study.

6. Analysis: The description of analysis is unclear – what does bottom-up from the full qual text mean? I also wonder what about the analysis was inductive? Though the authors say it was inductive-deductive – this is not at all clear. It feels very much that analysis was weighted towards a deductive analysis and the CFIR framework was imposed on the data with little of the conceptual or deeper thought usually characteristic of qualitative analysis

Response: Although we, in good-faith, perceived our study as using a combined inductive-deductive analysis method we can appreciate how the reviewer might disagree and argue it is mostly a deductive analysis. We have therefore changed the text to reflect this as follows

The full-text data was analyzed using thematic content analysis in a deductive approach according to Braun and Clarke's six-phase guide for thematic analysis in qualitative research.[25] Initially, the barriers and facilitators as expressed by the

participants were identified into codes.

Reviewer 2:

1. Title submitted and title in manuscript are different

Response: Thank you for, this notification. It has been corrected.

2. Introduction – more background into the types of procedures or patients where music interventions have been shown to be effective would be helpful. Is there evidence for minor procedures too? Also a brief description of music interventions would be helpful for those not familiar with it (e.g. is it just playing some music in the background, are there certain types of music used or can patients choose, etc).

Response: also see response to reviewer 1: We acknowledge that not every reader is as familiar with music interventions (as we are). We have expanded the introduction to include some more background information in music interventions and included an example of how clinical studies with music interventions are done. We hope this suffices.

3. Methods (study population) – there seems to be different recruitment strategies for greater Rotterdam and the rest of Netherlands. Was there a bias in the response based on region and would this have any implication on the results (i.e. do you expect differences in barriers and facilitators by region)?

Response: We did not observe a bias in response from participants from different regions nor was this expected. The Netherlands is a small and compact country where medical practices and guidelines are nationalized.

4. Page 9 lines 32-37 (last sentence of data collection section) – this relates to the process domain of CFIR, which was excluded. It's also not in the interview guide. Pls clarify if this was part of the interview and the purpose of this since the process domain was excluded.

Response: we thank the reviewer for this sharp observation; this question was not part of the interview and/or analysis. We have removed the line.

5. Supplementary table 1 can be in the main text

Response: changed accordingly (If the editor believes this table takes up too much space we have not problems moving it back to the supplementary files.

6. There’s no supplementary file 3 but there’s a file 4. Pls double check numbering.

Response: corrected

VERSION 3 - REVIEW

**Reviewer**	**2**
**Name**	**Chan, Sze Ling**
**Affiliation**	**Singhealth, Health Services Research Centre**
**Date**	**03-Jun-2026**
**COI**	

---

1. In Table 1, instead of giving examples in the third column, state what it is/how it is defined for this study. E.g. innovation = music interventions, inner setting = GP clinics, etc

2. On my previous comment on potential bias in response by region, the issue is more with whether the different recruitment strategies resulted in different types of respondents (e.g. more vocal/tend to face more barriers in those recruited through social media). The sentence “We did not expect differences in barriers and facilitators for this research subject based on region in The Netherlands.” can be removed from the methods. Rather, add this as a limitation and state that you did not observe a difference in responses by region and therefore the different recruitment strategies unlikely led to any bias.

VERSION 3 - AUTHOR RESPONSE

Reviewer: 2
Dr. Sze Ling Chan, Singhealth, Duke-NUS Graduate Medical School
Comments to the Author:
1. In Table 1, instead of giving examples in the third column, state what it is/how it is defined for this study. E.g. innovation = music interventions, inner setting = GP clinics, etc
2. On my previous comment on potential bias in response by region, the issue is more with whether the different recruitment strategies resulted in different types of respondents (e.g. more vocal/tend to face more barriers in those recruited through social media). The sentence “We did not expect differences in barriers and facilitators for this research subject based on region in The Netherlands.” can be removed from the methods. Rather, add this as a limitation and state that you did not observe a difference in responses by region and therefore the different recruitment strategies unlikely led to any bias.
